# Epithelial ovarian cancer survival by race and ethnicity in an equal-access healthcare population

**DOI:** 10.1038/s41416-023-02471-z

**Published:** 2023-12-06

**Authors:** Zhaohui L. Arter, Daniel Desmond, Jeffrey L. Berenberg, Jeffrey L. Killeen, Kristen Bunch, Melissa A. Merritt

**Affiliations:** 1 University of California, Irvine Medical Center, Irvine, CA USA; 2 Walter Reed National Military Medical Center, Bethesda, MD USA; 3 Tripler Army Medical Center, Honolulu, HI USA; 4 University of Hawaii Cancer Center, Honolulu, HI USA; 5 Department of Pathology, John A. Burns School of Medicine, University of Hawaii, Honolulu, HI USA; 6 Kapiolani Medical Center for Women and Children, Honolulu, HI USA; 7 The Daffodil Centre, The University of Sydney, a joint venture with Cancer Council New South Wales, Sydney, NSW Australia

**Keywords:** Translational research, Disease-free survival

## Abstract

**Background:**

Previous studies in the general population observed that compared with non-Hispanic White women, Pacific Islander and Black women have higher age-adjusted mortality rates from epithelial ovarian cancer (EOC), while Asian American patients have lower mortality. We investigated whether race and ethnicity is associated with differences in EOC survival in a United States Military population where patients have equal access to healthcare.

**Methods:**

This retrospective study included women diagnosed with EOC between 2001 and 2018 among Department of Defense beneficiaries. Hazard ratios (HRs) and 95% confidence intervals (CIs) were calculated using Cox proportional hazards regression models adjusting for age and year of diagnosis, histology and stage.

**Results:**

In our study population of 1230 invasive EOC cases (558 non-Hispanic White, 74 non-Hispanic Black, 73 Asian, 30 Pacific Islander and 36 Hispanic cases), 63% of the women died (all-cause death) after a mean = 4.8 years (SD = 4.1) of follow-up following diagnosis. Compared with non-Hispanic White cases, Asian cases had better overall survival, HR = 0.76 (95% CI = 0.58–0.98), whereas there were no differences in survival for other racial and ethnic groups.

**Conclusions:**

These findings highlight the need to investigate how differences in access to healthcare may influence observed racial and ethnic disparities for EOC.

## Introduction

Ovarian cancer is the second most prevalent gynaecologic cancer and the most lethal gynaecologic malignancy among women. Ovarian cancer survival rates are poor; only 50% of ovarian cancer patients in the United States survived for 5 years following diagnosis (2012–2018) [1], and only 31% of women diagnosed with distant invasive epithelial ovarian cancer (EOC) survived for 5 years [2]. EOC accounts for 95% of all ovarian cancer diagnoses; the main histologic subtypes include serous, endometrioid, clear cell, and mucinous subtypes. Compared to non-Hispanic White (hereafter referred to as White) women in the United States, Black women have the lowest EOC 5-year relative survival rate [1]. There are few data on EOC survival among other less common racial and ethnic groupings, although there is evidence that Hispanic and Asian women have a higher survival rate than White and Black women [3, 4]. Although variations in ovarian cancer survival by race are likely due to multiple factors, unequal access to healthcare was believed to play a significant role [5].

It is uncertain whether having equal access to the healthcare system, irrespective of race and ethnicity or socioeconomic background, will remove the overall disparity in EOC survival. The United States Department of Defense (DoD) Military Health System provides all beneficiaries with equal access to care. In a recent study utilising the DoD’s cancer registry system (Automated Central Tumor Registry or ACTUR database), Asian women with ovarian cancer were more likely than White women to receive care based on clinical practice guidelines [6]. There were no other noticeable differences in minority women’s receipt of recommended medicines compared to White women, however, the overall survival patterns were not studied. Another study conducted by Kaiser Permanente Northern California revealed that Black patients with invasive EOC had the lowest survival rate compared to White patients [7]. However, in the Kaiser health system, a patient must pay for a variety of medical procedures up front until the deductible is met, and even then, a substantial copayment is required for some appointments [8]. In contrast, these types of costs are completely covered under the Military Health System.

To our knowledge, this study is the first and largest ovarian cancer study to analyse overall survival data in EOC patients in an equitable access health system, extending the analysis to include other racial and ethnic groups (i.e., Pacific Islander) that have not been assessed previously. Findings from this study will provide new evidence to determine whether survival differences by race and ethnicity persist when EOC patients have equal access to healthcare.

## Materials and methods

### Study population

The current study population uses data extracted from the United States DoD ACTUR database which includes information on DoD beneficiaries, including active-duty military personnel, retirees and their dependents, who are diagnosed with cancer and receive cancer treatment at military facilities [9]. Military treatment facilities are mandated to report cancer cases to the ACTUR [10]. Local tumour registrars abstract and enter data for newly diagnosed cancer patients in consultation with gynaecologic oncologists. Ovarian cancer was defined as International Classification of Diseases for Oncology 2nd or 3rd revision codes C56, C57 and C48.1–C48.2 for ovarian, fallopian tube and primary peritoneal sites, respectively. This study included ovarian cancer cases that were diagnosed between 2001 and 2018. Data were obtained from the ACTUR database on tumour histology, stage [local, regional, distant or unknown by combining Surveillance, Epidemiology and End Results (SEER) summary stage variables], grade [well (G1), moderate (G2), poor (G3), undifferentiated (G4) or unknown], information on only the first course of treatment [surgery (yes/no); receipt of adjuvant chemotherapy (yes/no)] and vital status. Racial and ethnic groups were recorded in the registry database using: (1) information that was documented in the beneficiary medical record [which includes health data from the DoD, Department of Veterans Affairs and private sector partners) and outside/community records or documents]; (2) observations made by the treating/managing physicians; or (3) information obtained directly from the patient’s response to the Oncology/Registry Patient Questionnaire (when applicable). Individuals who reported more than one race and ethnicity (Asian, Black, Hispanic, White) were classified as more than one race and ethnicity; an exception was for Pacific Islander participants who were always classified in the Pacific Islander group (even if they reported more than one race and ethnicity).

We identified *N* = 1658 ovarian cancer cases and the following exclusions were applied: not invasive (*N* = 104); non-epithelial cases (*N* = 238, detailed in Supplementary Table 1); not in the five major racial and ethnic groups (Asian, Black, Hispanic, White, Pacific Islander) that were the focus of this study (*N* = 80); unknown vital status (*N* = 3); and date of diagnosis was the same as date of death (*N* = 3). This left *N* = 1230 cases for this study. The outcome of interest was all-cause mortality. Information on cause of death was not available for the majority of the patients. This study was approved by the Tripler Army Medical Center committee as Exempt Human Subjects research.

### Statistical analysis

HRs and 95% CIs were calculated using Cox proportional hazards regression models. Person-time was calculated as the number of days between a patient’s date of diagnosis until the date of last contact or censoring, whichever occurred first. Multivariable models were adjusted for covariates selected a priori because of their known influence on risk of EOC death; histology [serous (reference), endometrioid, clear cell, mucinous, not otherwise specified (other)] and stage [local (reference), regional, distant, missing]. We also adjusted for year of diagnosis (continuous) to account for possible changes in treatment over time. Age at diagnosis (continuous) was included as a strata term in all models. We tested whether additional adjustment for any of the following variables (grade; first-line treatment with surgery and/or chemotherapy; and residual disease) changed the risk estimates by 10% or more and the results were very similar therefore these variables were not adjusted for in the final models. The proportional hazards assumption was tested using the method described by Grambsch and Therneau, 1994 [11]; no violation of proportional hazards was observed. For the descriptive analyses of the population characteristics, age-standardised (indirectly standardised means and percentages) were calculated using 10-year age groups (age at diagnosis: <40 years, 40–49, 50–59, 60–69, 70 +).

We explored whether there were differences in survival by racial and ethnic group in subgroups by age at diagnosis (< 65 years, 65+ years) because older patients may be less likely to receive standard treatments and/or to develop toxicity [12]. We also carried out sensitivity analyses after restricting analyses to more homogeneous case subgroups: patients with high grade (grade 2 and higher) serous disease, those with regional/distant disease and patients who received a uniform first-line treatment (both surgery and chemotherapy). All statistical tests were two-sided and *P* values < 0.05 were considered statistically significant. Statistical analyses were performed using the survival package [13] in R version 4.1.0 [14].

## Results

After a mean follow-up of 4.8 (SD = 4.1) years, 63% of the *N* = 1230 invasive EOC cases were fatal (death from all causes) (Table 1). In a comparison of the age-standardised characteristics of EOC patients by racial and ethnic subgroups, we observed that a higher proportion of Asian cases had EOC from a fallopian tube site (13.7% versus ≤5.8% fallopian tube site in other groups) and a high proportion of Hispanic cases had a primary peritoneal tumour site (18.2% versus ≤7.4% in other groups). There were differences in the distribution of histological subtypes across racial and ethnic groups. Specifically, the proportion of serous tumours was highest in White, Black and Hispanic cases (≥ 56.0%) followed by Asian (49.7%) and Pacific Islander (42.1%) cases. The proportion of endometrioid tumours was highest in Pacific Islander cases (14.9%) compared with ≤10.1% in other groups. Asian cases had the highest proportion of clear cell EOC (10.5%) compared with ≤7.1% in other groups. A high proportion of Pacific Islander patients (69.1%) were diagnosed with distant-stage disease, followed by 58.6% of Asian patients and ≤55.8% among other groups. However, a higher proportion of Pacific Islander patients had low-grade/Grade 1 disease (19.0%) compared with other groups (≤ 12.9% Grade 1). In relation to the treatment received, a high proportion of Asian patients received both surgery and chemotherapy (79.0%) compared with ≤71.9% in other groups. A high proportion of Hispanic women had suboptimal residual disease following surgery (48.4%), followed by 38.0–38.7% of Asian and Black patients and ≤34.8% of Pacific Islander and White patients; however, a large proportion of patients (40.4% of all EOC cases) were missing information on residual disease.

**Table 1:** Age-standardised characteristics of epithelial ovarian cancer cases (*N* = 1230) by racial and ethnic groups in the ACTUR database.

		Racial and ethnic groups^a^				
	Total (*n* = 1230)	White (*n* = 853)	Black (*n* = 120)	Asian (*n* = 134)	Pacific Islander (*n* = 58)	Hispanic (*n* = 65)
Fatal (all causes), %	62.7	64.6	61.1	56.4	56.1	61.0
Age at diagnosis (years)^b^	57.5 (14.4)	58.3 (14.7)	58.0 (14.2)	55.3 (12.8)	55.2 (12.5)	52.8 (14.2)
Duration of follow-up (years)^b^	4.8 (4.1)	4.7 (4.1)	4.6 (3.7)	5.5 (4.5)	3.6 (3.7)	5.6 (4.2)
Tumour site: ovary, %	86.6	87.0	89.4	84.5	91.1	78.4
Tumour site: fallopian tube, %	6.5	5.7	5.1	13.7	5.8	3.4
Tumour site: primary peritoneal, %	6.9	7.4	5.5	1.8	3.1	18.2
Serous, %	55.4	56.0	60.1	49.7	42.1	56.5
Endometrioid, %	8.0	8.0	6.0	10.1	14.9	4.7
Clear cell, %	7.1	7.1	5.3	10.5	5.8	5.9
Mucinous, %	7.2	7.3	5.4	7.2	6.5	7.4
NOS histology, %	15.9	15.9	14.7	18.3	21.7	13.1
Other histology, %	6.3	5.7	8.4	4.2	8.9	12.5
Local stage, %^c^	19.5	19.8	21.0	14.1	18.7	19.9
Regional stage, %^c^	26.4	27.0	26.0	27.3	12.2	24.3
Distant stage, %^c^	54.1	53.2	52.9	58.6	69.1	55.8
Grade 1, %^d^	12.8	12.9	10.9	12.4	19.0	11.8
Grade 2, %^d^	15.0	14.8	21.6	9.2	15.9	13.7
Grade 3, %^d^	60.8	61.9	56.6	60.9	44.4	64.7
Grade 4, %^d^	11.5	10.4	10.9	17.5	20.8	9.8
First course of treatment						
No surgery or chemotherapy, %^c^	3.9	3.7	3.9	4.1	5.9	4.2
Surgery and chemotherapy, %^c^	70.8	69.5	70.5	79.0	69.3	71.9
Chemotherapy only, %^c^	7.0	6.6	8.5	6.6	15.2	7.0
Surgery only, %^c^	18.3	20.2	17.0	10.3	9.6	16.9
Surgical outcome						
Optimal residual disease, %^e^	65.2	66.1	61.3	62.0	72.4	51.6
Suboptimal residual disease, %^e^	34.8	33.9	38.7	38.0	27.6	48.4

Values are means (SD) for continuous variables or percentages for categorical variables and are standardised to the age distribution of the study population.

^a^Asian includes Chinese, Japanese, Filipino, Korean and Vietnamese cases. Pacific Islander includes Hawaiian, Micronesian, Guamanian and Samoan cases.

^b^Value is not age-standardised.

^c^Data on stage and first course of treatment were missing for 4.4% and 2.9% of cases, respectively.

^d^Grade was missing for 32.0% overall (missing for 30.5% of non-Hispanic White, 30.1% of Black, 33.8% of Asian, 51.3% of Pacific Islander and 39.4% of Hispanic cases).

^e^Residual disease was missing for 40.4% overall (missing for 41.3% of non-Hispanic White, 42.5% of Black, 39.9% of Asian, 38.3% of Pacific Islander and 32.7% of Hispanic cases).

Among all EOC cases, we observed that compared with White cases, Asian cases had improved survival, HR = 0.76 (95% CI = 0.58–0.98) while there was no difference in survival for other racial and ethnic groups: Black, HR = 1.08 (95% CI = 0.83–1.40); Pacific Islander, HR = 1.19 (95% CI = 0.80–1.76); and Hispanic cases, HR = 0.81 (95% CI = 0.56–1.17) (Table 2). In further sensitivity analyses restricting to patients who received a uniform treatment (chemotherapy and surgery), there was a similar improved survival for Asian patients HR = 0.70 (95% CI = 0.52–0.95) as compared with White patients. When restricting to cases with regional/distant disease, the improved survival observed among Asian cases was attenuated [Asian compared with White cases, HR = 0.77 (95% CI = 0.58–1.01)]. In sensitivity analysis of the most common histologic subtype of EOC (serous high grade), there were no differences in survival across racial and ethnic groups.

**Table 2:** Association between racial and ethnic groups and survival among epithelial ovarian cancer overall and for selected case groups.

Epithelial ovarian cancer groups	Racial and ethnic groups	Total	Fatal cases (%)	HR (95% CI)
Total epithelial ovarian cancer	White	853	558 (65.4%)	1.00 (Ref)
*N* = 1230	Black	120	74 (61.7%)	1.08 (0.83–1.40)
	Asian	134	73 (54.5%)	**0.76 (0.58–0.98)**
	Pacific Islander	58	30 (51.7%)	1.19 (0.80–1.76)
	Hispanic	65	36 (55.4%)	0.81 (0.56–1.17)
Serous high grade (G2-4)	White	324	237 (73.1%)	1.00 (Ref)
*N* = 461	Black	52	39 (75.0%)	0.84 (0.56–1.26)
	Asian	48	33 (68.8%)	0.81 (0.53–1.23)
	Pacific Islander	17	13 (76.5%)	1.53 (0.81–2.89)
	Hispanic	20	10 (50.0%)	0.82 (0.40–1.68)
Regional/distant disease	White	659	486 (73.7%)	1.00 (Ref)
*N* = 947	Black	90	64 (71.1%)	1.06 (0.79–1.42)
	Asian	111	69 (62.2%)	0.77 (0.58–1.01)
	Pacific Islander	41	21 (51.2%)	0.90 (0.56–1.45)
	Hispanic	46	27 (58.7%)	0.73 (0.47–1.11)
Cases received chemotherapy and surgery	White	571	380 (66.5%)	1.00 (Ref)
*N* = 845	Black	82	52 (63.4%)	1.02 (0.74–1.41)
	Asian	105	54 (51.4%)	**0.70 (0.52–0.95)**
	Pacific Islander	40	19 (47.5%)	1.01 (0.61–1.67)
	Hispanic	47	25 (53.2%)	0.81 (0.52–1.26)

*CI* confidence interval, *G2-4* tumour grades 2–4, *HR* hazard ratio.

Multivariable models were adjusted for histology [serous (reference), endometrioid, clear cell, mucinous, not otherwise specified [NOS], other], stage [local (reference), regional, distant, missing] and year of diagnosis (continuous); age at diagnosis (continuous) was modelled as a strata term.

Bold values indicate that the association is statistically significant.

We conducted exploratory analyses considering age at diagnosis to account for possible differences in treatment response and observed no heterogeneity in associations between different racial and ethnic groups with mortality risk across the two age groups (split at age 65 years) (*P*-heterogeneity=0.33) (Table 3). Notably, the improved survival for Asian patients was only apparent among cases who were aged <65 years at diagnosis [Asian compared with White cases, <65 years at diagnosis, HR = 0.67 (95% CI = 0.49–0.92); 65+ years, HR = 0.98 (95% CI = 0.60–1.58)]. There was also a suggestion that Black patients who were diagnosed at age 65+ years may have a higher mortality risk [Black compared with White cases, <65 years at diagnosis, HR = 0.89 (95% CI = 0.63–1.25); 65+ years, HR = 1.45 (95% CI = 0.93–2.25)].

**Table 3:** Association between racial and ethnic groups and survival among all epithelial ovarian cancer cases splitting age at diagnosis groups at 65 years.

	Age < 65 years			Age 65+ years			
Racial and ethnic groups	Total	Fatal cases (%)	HR (95% CI)^a^	Total	Fatal cases (%)	HR (95% CI)^a^	*P*-heterogeneity
White	576	331 (57.5%)	1.00 (Ref)	277	227 (81.9%)	1.00 (Ref)	0.33
Black	82	44 (53.7%)	0.89 (0.63–1.25)	38	30 (78.9%)	1.45 (0.93–2.25)	
Asian	104	51 (49.0%)	**0.67 (0.49–0.92)**	30	22 (73.3%)	0.98 (0.60–1.58)	
Pacific Islander	45	19 (42.2%)	1.11 (0.67–1.81)	13	11 (84.6%)	1.14 (0.58–2.25)	
Hispanic	55	27 (49.1%)	0.79 (0.52–1.20)	10	9 (90.0%)	0.94 (0.45–1.99)	

*CI* confidence interval, *HR* hazard ratio.

^a^Adjusted for histology [serous (reference), endometrioid, clear cell, mucinous, not otherwise specified [NOS], other], stage [local (reference), regional, distant, missing] and year of diagnosis (continuous); age at diagnosis (continuous) was modelled as a strata term.

Bold values indicate that the association is statistically significant.

## Discussion

Numerous studies utilising population-based databases have uncovered racial, ethnic, and socioeconomic inequities in ovarian cancer care and treatment access [7, 15–17]. Our goal in the current study was to assess risk of EOC mortality using data from patients treated in the Military Health System where access to healthcare is universal. We observed that there were no differences in EOC survival between White patients and Black patients. Notably, Asian patients had a lower mortality risk compared with White patients.

Our observation of no difference in survival between White patients and Black patients in a Military Health System contrasts with a previous report using SEER data from 1995 to 2015 which showed that Black EOC patients were at higher risk of all-cause mortality (HR 1.28, 95% CI 1.23–1.33) than White patients in models that were adjusted for age at diagnosis, stage, grade, subtype, surgical intervention, chemotherapy, radiation, laterality, insurance status and SEER registry region [18]. In the most recent US cancer statistics report using SEER data, the 5-year relative survival rate for Black women with EOC was the lowest (41%) compared to White women (49%) [1]. Albain et al. [19] similarly observed a 10-year survival rate of 13% for Black women versus 17% for all other patients with advanced stage (Stage III or IV) ovarian cancer using data from randomised clinical trials patients of the Southwest Oncology Group. In contrast to the observed disparities in survival reported for Black women with EOC using general population data, our findings using data from the Military Health System support the idea that survival disparities could be largely influenced by access to healthcare.

We observed that Asian patients had a lower mortality risk compared with White patients. This result was consistent with the Fuh et al. [3] study using SEER data, where it showed that the 5-year disease-specific survival of Asian patients with EOC was higher compared to White patients (59.1% vs. 47.3%, respectively, *P* = 0.001). In a meta-analysis of EOC patients who enrolled in 10 Gynaecologic Oncology Group clinical trials there was a similar improvement in disease-specific survival in Asian patients (*N* = 273) compared with White patients (*N* = 7641) (Asian compared with White patients, HR 0.84, 95% CI 0.72–0.99) after accounting for age, body mass index, better performance status, stage, histology, grade and residual disease [20]. This study did not include other racial and ethnic groups.

The current report showed differences in the distribution of histological subtypes across racial and ethnic groups. Specifically, Asian cases had the highest proportion of clear cell EOC (10.5%) compared with ≤7.1% in other groups. This finding is consistent with a report from Park et al. who observed that a higher proportion of Asian EOC patients were diagnosed with clear cell tumours (11.7%) than other racial and ethnic groups (clear cell tumours ranging from 2.4% to 4.5%), with Black women the least likely to be diagnosed with clear cell EOC (2.4%) [4]. We found that the proportion of serous tumours was highest among White, Black and Hispanic patients (≥ 56%), followed by Asian (49.7%) and Pacific Islander (42.1%) patients. A prior study also found serous cases to be the least frequently diagnosed among Asian women, while White and Black women had equivalent proportions of serous EOC diagnoses [4]. Considering that treatment techniques are not uniformly effective across EOC histotypes [21–23], variations in histologic subtype distribution by race may contribute to racial and ethnic survival differences. We accounted for differences in histologic subtype proportion by adjusting for histology in the multivariable models.

The standard recommended treatment for ovarian cancer by the National Comprehensive Cancer Network (NCCN) is cytoreductive surgery along with platinum- and taxane-based chemotherapy [24]. Eaglehouse et al. showed that Asian women were > 2 times as likely to seek NCCN guideline-based care than White women in a recent study employing ACTUR data as well as the Military Health System Data Repository administrative claims data [6]. In our study, we found that 79.0% of Asian patients underwent both surgery and chemotherapy, compared to 71.9% of patients in other racial and ethnic groups. Importantly the improvement in survival among Asian individuals was still apparent when we restricted the analyses to cases who received uniform treatment (chemotherapy and surgery). This result suggests that factors other than receipt of treatment may explain the improved survival in Asian EOC patients. It was suggested that women with *BRCA* germline mutations have higher response rates to both platinum- and nonplatinum-based regimens than mutation-negative patients [25], and certain Asian groups were found to have a higher predisposition to *BRCA* mutations such as the Chinese women from Hong Kong and Korea [26, 27]. *BRCA* mutation status was not available in the current study. It will be of interest to consider BRCA mutation status (germline and tumour somatic mutations) in future studies focusing on racial and ethnic differences in EOC survival.

Our study had several strengths, including the ability to evaluate EOC overall survival in a DoD Military Health System with equal access to free medical care. Another strength was that we included Pacific Islander and Hispanic EOC patients who have not been included in earlier studies focusing on EOC survival. There are also some limitations of this study including the lack of information on treatment data beyond the first primary treatment. We also lacked information on the cause of death however we anticipate that our results for all-cause mortality will be mostly congruent with findings for EOC-specific death, given that EOC is a highly aggressive disease, and consequently the majority of deaths in this patient cohort will be attributable to EOC or its sequelae. It is possible that some women who are diagnosed in the military, and tracked by the ACTUR registry, received some of their care outside the military health system, including some academic facilities. This could explain why data on the first course of treatment (< 5%) were missing. In the ACTUR database, there were three methods of race and ethnicity reporting; two of the methods were based on self-report from the patient while the third method involved physician reports. Physician-reported race and ethnicity is less reliable than self-reports from the patients themselves. This could lead to misclassification of race and ethnicity and may attenuate risk estimates towards the null value. Our study included TRICARE recipients inside the Military Health System, which may not be representative of the racial and ethnic composition of the overall United States population. Although our study was large, the number of patients did not allow further subgroup stratification (e.g., consideration of Chinese, Korean, Filipino subgroups). SEER statistics indicate that Asian subgroups have varying 5-year ovarian cancer survival rates, ranging from 62.1% for Vietnamese to 48.2% for Asian Indian/Pakistani [3].

In summary, uneven access to care is hypothesised to play a significant role in the observed racial and ethnic disparities in EOC survival rates. Thus our goal was to determine if race and ethnicity are associated with variations in EOC survival in a military population with equal access to healthcare. With the exception of a slight survival advantage for Asian patients, we observed no racial or ethnic differences in EOC survival for Black, Pacific Islander and Hispanic patients as compared with White patients. These results underscore the need to investigate how differences in access to healthcare may influence observed racial and ethnic disparities for EOC.

## Disclaimer

The contents of this publication are the sole responsibility of the author(s) and do not necessarily reflect the views, opinions, or policies of Uniformed Services University of the Health Sciences (USUHS), the Department of Defense (DoD) or the Departments of the Army, Navy, or Air Force.

### Supplementary information

Supplemental Table 1

## Data Availability

This study used data from the United States Department of Defense Automated Central Tumor Registry (ACTUR) database which are not publically available due to privacy concerns. Data described in the manuscript, code book, and analytic code are available from the corresponding author upon request.

## Code availability

Data described in the manuscript, code book, and analytic code are available from the corresponding author upon request.

## References

[1] Siegel RL, Miller KD, Wagle NS, Jemal A (2023). Cancer statistics, 2023. CA Cancer J Clin.

[2] SEER cancer statistics. Surveillance Research Program, National Cancer Institute. [Internet]. [cited February 23, 2023]. Available from https://seer.cancer.gov/explorer/

[3] Fuh KC, Shin JY, Kapp DS, Brooks RA, Ueda S, Urban RR (2015). Survival differences of Asian and Caucasian epithelial ovarian cancer patients in the United States. Gynecol Oncol.

[4] Park HK, Ruterbusch JJ, Cote ML (2017). Recent trends in ovarian cancer incidence and relative survival in the United States by race/ethnicity and histologic subtypes. Cancer Epidemiol Biomark Prev.

[5] Terplan M, Smith EJ, Temkin SM (2009). Race in ovarian cancer treatment and survival: a systematic review with meta-analysis. Cancer Causes Control.

[6] Eaglehouse YL, Darcy KM, Tian C, Casablanca Y, Shriver CD, Zhu K (2021). Racial-ethnic comparison of guideline-adherent gynecologic cancer care in an equal-access system. Obstet Gynecol.

[7] Bandera EV, Lee VS, Rodriguez-Rodriguez L, Powell CB, Kushi LH (2016). Racial/ethnic disparities in ovarian cancer treatment and survival. Clin Cancer Res.

[8] Plans KPD. Available from https://healthy.kaiserpermanente.org/shop-plans/deductible-plans 2019.

[9] Zheng L, Enewold L, Zahm SH, Shriver CD, Zhou J, Marrogi A (2012). Lung cancer survival among black and white patients in an equal access health system. Cancer Epidemiol Biomark Prev.

[10] Lin J, Hu H, Shriver CD, Zhu K (2022). Survival among breast cancer patients: comparison of the U.S. military health system with the surveillance, epidemiology and end results program. Clin Breast Cancer.

[11] Grambsch P, Therneau T (1994). Proportional hazards tests and diagnostics based on weighted residuals. Biometrika..

[12] Tew WP, Muss HB, Kimmick GG, Von Gruenigen VE, Lichtman SM (2014). Breast and ovarian cancer in the older woman. J Clin Oncol.

[13] Therneau TA. Package for survival analysis in S. R package version 32-11. [Internet]. 2021 9/12/2021. Available from http://CRAN.R-project.org/package=survival.

[14] R Core Team. R: a language and environment for statistical computing. Vienna, Austria: R Foundation for Statistical Computing; 2022 [updated 7/21/2022. Available from http://www.R-project.org/.

[15] Bristow RE, Powell MA, Al-Hammadi N, Chen L, Miller JP, Roland PY (2013). Disparities in ovarian cancer care quality and survival according to race and socioeconomic status. J Natl Cancer Inst.

[16] Peterson CE, Rauscher GH, Johnson TP, Kirschner CV, Barrett RE, Kim S (2014). The association between neighborhood socioeconomic status and ovarian cancer tumor characteristics. Cancer Causes Control.

[17] Terplan M, Schluterman N, McNamara EJ, Tracy JK, Temkin SM (2012). Have racial disparities in ovarian cancer increased over time? An analysis of SEER data. Gynecol Oncol.

[18] Stenzel AE, Buas MF, Moysich KB (2019). Survival disparities among racial/ethnic groups of women with ovarian cancer: an update on data from the surveillance, epidemiology and end results (SEER) registry. Cancer Epidemiol.

[19] Albain KS, Unger JM, Crowley JJ, Coltman CA, Hershman DL (2009). Racial disparities in cancer survival among randomized clinical trials patients of the Southwest Oncology Group. J Natl Cancer Inst.

[20] Fuh KC, Java JJ, Chan JK, Kapp DS, Monk BJ, Burger RA (2019). Differences in presentation and survival of Asians compared to Caucasians with ovarian cancer: an NRG Oncology/GOG Ancillary study of 7914 patients. Gynecol Oncol.

[21] Brown J, Frumovitz M (2014). Mucinous tumors of the ovary: current thoughts on diagnosis and management. Curr Oncol Rep.

[22] Takano M, Tsuda H, Sugiyama T (2012). Clear cell carcinoma of the ovary: is there a role of histology-specific treatment?. J Exp Clin Cancer Res.

[23] Shida D, Takabe K, Kapitonov D, Milstien S, Spiegel S (2008). Targeting SphK1 as a new strategy against cancer. Curr Drug Targets.

[24] Oncology NCCNCPGi. Available from https://www.nccn.org/ 2023.

[25] Alsop K, Fereday S, Meldrum C, deFazio A, Emmanuel C, George J (2012). BRCA mutation frequency and patterns of treatment response in BRCA mutation-positive women with ovarian cancer: a report from the Australian Ovarian Cancer Study Group. J Clin Oncol.

[26] Kwong A, Ng EK, Wong CL, Law FB, Au T, Wong HN (2012). Identification of BRCA1/2 founder mutations in Southern Chinese breast cancer patients using gene sequencing and high resolution DNA melting analysis. PLoS ONE.

[27] Noh JM, Choi DH, Baek H, Kim MJ, Park H, Huh SJ (2014). Genetic anticipation of familial breast cancer with or without BRCA mutation in the Korean population. Cancer Genet.

