# Supplementary material for: Epithelial ovarian cancer survival by race and ethnicity in an equal-access healthcare population
Source: Br J Cancer. 2023 Dec 6;130(1):108–13. doi: 10.1038/s41416-023-02471-z (PMC10781944; doi:10.1038/s41416-023-02471-z)
Supplement: Supplementary file 1 — Supplemental Table 1 [file 41416_2023_2471_MOESM1_ESM.docx]

Arter *et al*. ‘Epithelial ovarian cancer survival by race and ethnicity in an equal-access healthcare population’

**Supplementary tables**

**Supplementary Table 1**. International Classification of Diseases for Oncology 2nd or 3rd revision (ICD-O2 and -3, respectively) topography and morphology codes used to define invasive epithelial ovarian cancer and histological subtypes in the U.S. Department of Defense's Automated Central Tumor

Registry.

| **ICD-O2/-O3 topography code**  Include*:* C56, C57, C48.1-C48.2 | **ICD-O2/-O3 morphology codes**  Exclude*:* 8000, 8031, 8041, 8045, 8070, 8076, 8243, 8246, 8320, 8340, 8490, 8620, 8631, 8634, 8670, 8804, 8806, 8890, 8900, 8920, 8930, 8933, 8936, 9050, 9052, 9053, 9060, 9064, 9071, 9080, 9085, 9090, 9100, 9101, 9591, 9680, 9687, 9690, 9691, 9698, 9930 |
| --- | --- |

| **Tumor histology classification** | **ICD-O2/-O3 morphology codes** | **Total *N*=1230** |
| --- | --- | --- |
| Serous | 8020 | 5 |
|  | 8021 | 3 |
|  | 8050 | 5 |
|  | 8120 | 5 |
|  | 8260 | 6 |
|  | 8441 | 364 |
|  | 8450 | 2 |
|  | 8460 | 177 |
|  | 8461 | 113 |
|  | 9014 | 2 |
| Endometrioid | 8380 | 99 |
|  | 8570 | 0 |
| Clear cell | 8310 | 87 |
|  | 8313 | 0 |
| Mucinous | 8470 | 23 |
|  | 8471 | 1 |
|  | 8480 | 65 |
|  | 8481 | 0 |
| Adenocarcinoma not otherwise specified (NOS) | 8010 | 69 |
|  | 8140 | 119 |
|  | 8440 | 8 |
| Other specified epithelial ovarian cancer (including carcinosarcoma) | 8240 | 7 |
|  | 8255 | 4 |
|  | 8323 | 27 |
|  | 8950 | 14 |
|  | 8980 | 19 |
|  | 8951 | 4 |
|  | 9000 | 2 |
